# Synthesis of Gold Nanoparticles by Using Green Machinery: Characterization and In Vitro Toxicity

**DOI:** 10.3390/nano11030808

**Published:** 2021-03-22

**Authors:** Ahmed Al Saqr, El-Sayed Khafagy, Ahmed Alalaiwe, Mohammed F. Aldawsari, Saad M. Alshahrani, Md. Khalid Anwer, Salman Khan, Amr S. Abu Lila, Hany H. Arab, Wael A. H. Hegazy

**Affiliations:** 1 Department of Pharmaceutics, College of Pharmacy, Prince Sattam Bin Abdulaziz University, Al-kharj 11942, Saudi Arabia; a.alsaqr@psau.edu.sa (A.A.S.); a.alalaiwe@psau.edu.sa (A.A.); moh.aldawsari@psau.edu.sa (M.F.A.); sm.alshahrani@psau.edu.sa (S.M.A.); m.anwer@psau.edu.sa (M.K.A.); 2 Department of Pharmaceutics and Industrial Pharmacy, Faculty of Pharmacy, Suez Canal University, Ismailia 41522, Egypt; 3 Department of Biosciences, Integral University, Lucknow 226026, India; salmank@iul.ac.in; 4 Department of Pharmaceutics and Industrial Pharmacy, Faculty of Pharmacy, Zagazig University, Zagazig 44519, Egypt; a.abulila@uoh.edu.sa; 5 Department of Pharmacology and Toxicology, College of Pharmacy, Taif University, Taif 21944, Saudi Arabia; h.arab@tu.edu.sa; 6 Department of Microbiology and Immunology, Faculty of Pharmacy, Zagazig University, Zagazig 44519, Egypt; waelmhegazy@daad-alumni.de

**Keywords:** antibacterial, anticancer, auric chloride (gold salt), *Benincasa hispida*, gold nanoparticles (GNPs)

## Abstract

Green synthesis of gold nanoparticles (GNPs) with plant extracts has gained considerable interest in the field of biomedicine. Recently, the bioreduction nature of herbal extracts has helped to synthesize spherical GNPs of different potential from gold salt. In this study, a fast ecofriendly method was adopted for the synthesis of GNPs using fresh peel (aqueous) extracts of *Benincasa hispida*, which acted as reducing and stabilizing agents. The biosynthesized GNPs were characterized by UV–VIS and Fourier transform infrared spectroscopy, transmission electron microscopy (TEM), and dynamic light scattering. In addition, the in vitro antibacterial and anticancer activities of synthesized GNPs were investigated. The formation of gold nanoparticles was confirmed by the existence of a sharp absorption peak at 520 nm, corresponding to the surface plasmon resonance (SPR) band of the GNPs. TEM analysis revealed that the prepared GNPs were spherical in shape and had an average particle size of 22.18 ± 2 nm. Most importantly, the synthesized GNPs exhibited considerable antibacterial activity against different Gram-positive and Gram-negative bacteria. Furthermore, the biosynthesized GNPs exerted remarkable in vitro cytotoxicity against human cervical cancer cell line, while sparing normal human primary osteoblast cells. Such cytotoxic effect was attributed to the increased production of reactive oxygen species (ROS) that contributed to the damage of HeLa cells. Collectively, peel extracts of *B. hispida* can be efficiently used for the synthesis of GNPs, which can be adopted as a natural source of antimicrobial and anticancer agent.

## 1. Introduction

The increasing antimicrobial resistance has become one of the major public health challenges especially in the era of decreased discovery of new safe antimicrobial agents [1,2]. Several approaches have been exploited to renew the available antimicrobial chemotherapeutic options [3,4,5]. Currently, nanotechnology holds promise as an emerging technology for developing new antimicrobial agents with expanding properties such as efficient targeting, improvement of pharmacokinetic profile, and reduction of toxicity [5,6]. Gold nanoparticles (GNPs) are emerging materials that exhibit optical and electrical characteristics distinct from those of traditional materials and show promising potential for application in medicine [6,7]. These properties include high surface area to volume ratio, surface chemistry and multifunctionalization, surface plasmon resonance, and stable nature. Advantageously, GNPs can be easily synthesized into different shapes and sizes by fine tuning the components and concentrations [8]. The tunable size of GNPs eases their penetration through cellular membrane to affect metabolism, protein synthesis, and cellular permeability, resulting in bacterial cell death [6,9]. Many reports have recently emphasized the potential antibacterial efficacy of GNPs, besides their employment as carriers to antibodies, antibiotics, vaccines, and drugs [10,11,12,13]. There are several proposed mechanism of GNPs antimicrobial activities: (i) penetrative capability into microbial cells, (ii) augmenting cell membrane damage, (iii) aiding the disruption of bacterial DNA, and (iv) release of reactive oxygen species (ROS) [7,9,14].

Besides their antimicrobial activity, gold compounds have recently gained growing interest in the design of new metal anticancer drugs [15]. Of particular, gold nanoparticles have emerged as potential agents for cancer therapy and have been explored as drug carriers [16,17], contrast agents [18], photothermal agents [19,20], and radiosensitizers [21,22]. The nonimmunogenic and nontoxic nature and the better penetration ability than traditional drugs constitute merits that enable GNPs accumulation in the tumor sites. The GNPs anticancer activity is owed to their cytotoxic effects, inhibition of thiol-containing enzymes, particularly TrxR [23], damaging DNA [24] and mitochondrial functions [25]. Moreover, gold compounds can efficiently promote cellular mediated immune response against cancer through efficient antigen presentation on dendritic cells (DCs) [26]. Interestingly, there are other recognized antimicrobial and anticancer immune-regulatory effects of gold compounds that have been reviewed [27,28].

Despite the availability of versatile chemical and physical methods for the synthesis of nanoparticles, there is an increasing need to develop ecologically friendly methods to avoid the use of toxic chemicals, especially for medical purpose [29]. Plant-mediated biosynthesis of nanoparticles has emerged as an efficient environmentally friendly method for synthesis of metallic nanoparticles, alleviating the use of organic/toxic chemicals [30]. Synthesis of gold nanoparticles with plant extracts has gained great interest in the field of biomedicine due to its wide variety of health applications. Several approaches have been proposed to fabricate green, cost-effective, and controlled GNPs [31,32,33]. For instance, spherical crystalline pattern GNPs were prepared using aqueous extracts *of Salicornia brachiate*, which act as a catalyst in the synthesis process. The produced GNPs showed broad spectrum antibacterial activity against both Gram-positive and Gram-negative bacteria [31]. Similarly, gold nanoparticles (GNPs) were synthesized using *Mangifera indica* seed extract. The bioactive molecules in the seed act as a reducing agent. The green synthesized GNPs show efficient moderate antibacterial activity against different pathogenic bacteria, with only a moderate cytotoxic effect against the HeLa and MCF-7 breast cancer cell lines [32].

*Benincasa hispida*, commonly called winter melon or white pumpkin, is a member of the Cucurbitaceae family. It is well recognized with its nutritional and medicinal properties, particularly among Asian countries [34,35]. The main constituents of *B. hispida* fruits include volatile oils, glycosides, flavonoids, vitamins, and minerals. Traditionally, *B. hispida* had been used for the alleviation of various complaints such as respiratory disease, gastrointestinal problems, urinary diseases, and heart diseases [36]. In addition, many reports demonstrated that *B. hispida* exerts many neuropharmacological effects such as muscle relaxant, anxiolytic, antidepressant, as well as positive effects in psychological disorders such as epilepsy, dementia, and schizophrenia [37]. Recently, we utilized peel extract of *B. hispida* for the synthesis of silver nanoparticles [36]. This method enabled nanoparticle synthesis in a one-pot process. This is because peel extract of *B. hispida* acts not only as bioreducing agents but as stabilizing agents for the formed particles as well.

In this study, therefore, we aimed to green synthesize GNPs and evaluate their antimicrobial and anticancer activities. In this context, *B. hispida* aqueous peel extract has been used to synthesize GNPs. The antibacterial activity of GNPs was screened against different Gram-positive and Gram-negative bacterial strains. Furthermore, the cytotoxic and anticancer activities of synthesized GNPs were evaluated against normal osteoblast cells and human cervical cancer cells (HeLa cells).

## 2. Materials and Methods

### 2.1. Materials

Gold chloride (HAuCl_4_) was obtained from Sigma-Aldrich (St. Louis, MO, USA). *B. hispida* was acquired as industrial waste from a local sweet factory. 3-(4,5-dimethylthiazol-2-yl)-2,5-diphenyl tetrazolium bromide (MTT reagent), 2′,7′-dichlorodihydrofluorescein diacetate (H2DCFDA reagent), and Dulbecco’s modified Eagle medium (DMEM) were procured from Sigma-Aldrich (St. Louis, MO, USA). Müeller Hinton (MH) broth, agar, and tryptone soya broth (TSB) were procured from Himedia (Mumbai, India). All other solvents and chemicals were of analytical grade.

### 2.2. Bacterial Strains, Cell Lines, and Cultivation Conditions

*Staphylococcus aureus* (ATCC 25923), *Eschrichia coli* (ATCC 25922), *Salmonella abony* (ATCC BAA-2162), and *Klebsiella pneumonia* (ATCC 13883) were used to test the antibacterial activity of GNPs. The bacterial strains were cultivated and maintained at 37 °C on MH agar media. The normal human primary osteoblasts and human cervical cancer cells (HeLa) were supplied from National Centre for Cell Science (NCCS), Pune, India. The cell lines were grown as monolayer in MacCoy’s and DMEM media with supplementation of 10% fetal bovine serum and 1% actinomycin. The cell lines were subcultured and maintained under a humidified atmosphere at 37 °C.

### 2.3. Preparation of Fresh Aqueous Peel Extract of B. hispida

Fresh peel of *B. hispida* were collected, rinsed repeatedly with distilled water to remove any impurities from their surfaces. Then, the fresh peel was cut into small pieces, ground with double distilled water using pestle mortar placed in a tray filled with ice to avoid protein denaturation. The mixture was filtered using the Whatman^®^ Grade 42 filter paper followed by centrifugation at 6000 rpm for 15 min. The supernatant was collected, filtered and finally the aqueous was stored at 4 °C for further use in the synthesis of GNPs.

### 2.4. Biosynthesis of GNPs

Gold nanoparticles were synthesized by the reduction method using 1 mM aqueous gold salt solution. Briefly, equal volumes of gold salt solution and aqueous peel extracts were mixed together to give a final volume of 30 mL. The mixture was held at 40 °C for 24 h. Upon incubation, the solution color changes from light green to ruby red, indicating the completion of reaction. The mixture was then filtered using a 2 µm syringe filter and unbound proteins were separated by precipitation with absolute ethanol. Finally, the obtained gold nanoparticles were kept at 4 °C for further experiments.

### 2.5. Characterization of GNPs

#### 2.5.1. UV–Visible Spectroscopy

The reduction of gold salts into gold nanoparticles was confirmed by using dual-beam UV–VIS spectroscopy (Shimadzu dual-beam spectrophotometer UV-1601 PC Series, Shimadzu, Tokyo, Japan) operated at a resolution of 1 nm in the range of 200 to 800 nm. This technique depends on color change owing to the reduction of metal salts to biosynthesized gold nanoparticles.

#### 2.5.2. Particle Size and Zeta Potential

The hydrodynamic radius of the prepared nanoparticles was estimated by dynamic light scattering (DLS). Both particle size and zeta potential were measured using a Zetasizer Nano-ZS (ZEN3600 Malvern Instrument Ltd., Malvern, UK).

#### 2.5.3. Transmission Electron Microscopy (TEM)

Transmission electron microscopy was adopted to investigate the morphology and size of the gold nanoparticles. One drop of GNPs suspension was distributed onto carbon-coated TEM copper grids followed by analysis on a Tecnai G2 Spirit transmission electron microscope equipped with a BioTwin lens configuration (Hillsboro, OR, USA), operated at an accelerating voltage of 80 kV.

#### 2.5.4. Fourier Transform Infrared (FTIR) Spectroscopy

FTIR spectroscopy was used to trace the existence of different functional groups at the surface of GNPs. The spectrum was obtained by a Perkin-Elmer Spectrum FTIR system (PerkinElmer Inc., Waltham, MA, USA) at the range of 650–4000 cm^−1^.

### 2.6. Antibacterial Activity Evaluation of GNPs

The antibacterial activities of synthesized GNPs were evaluated against various Gram-negative and Gram-positive bacterial strains. Synthesized GNPs were dispersed in phosphate buffer saline (PBS) and the prepared solutions were used at pH 7.2.

#### 2.6.1. Qualitative Assessment of Antibacterial Activity

To determine the ability of synthesized GNPs to inhibit bacterial growth, the agar diffusion method was used according to the Clinical Laboratory and Standards Institute Guidelines (CLSI 2015) [38]. Standardized suspensions of the tested strains (equivalent to the 0.5 McFarland) were prepared from overnight cultures in TSB and swabbed over the surface of Müeller–Hinton agar plates. Equal amounts of GNPs (10 μg/mL) and PBS as negative control were added to the wells made in MH agar plates. The experiment was carried out in triplicate and the plates were incubated at 37 °C overnight and the diameters of inhibition zones were measured.

#### 2.6.2. Determination of Synthesized GNPs MIC and MBC Values

The minimum inhibitory concentrations (MICs) of synthesized GNPs against tested bacterial strains were determined using the broth microdilution method according to CLSI (2015) [4]. Aliquots of GNPs were serially diluted in 96-well microtiter plates containing TSB medium to achieve a range of concentrations (0.1–120 μg/mL). Aliquots (10 μL) from prepared standard suspensions of tested strains, which were cultured overnight in TSB and their optical densities were adjusted to OD600 of 0.4 (2 × 10^5^ CFU/mL), were added to each well. The MICs were the lowest concentrations of synthesized GNPs that completely inhibited the bacterial growth after incubation at 37 °C for 20 h and the bacterial cells were viably counted from each well. Meanwhile, the lowest concentrations of GNPs that showed no visible growth upon subculturing onto fresh medium were considered minimum bactericidal concentrations (MBCs) [38]. Colistin sulfate was used as a positive control, sterile PBS was involved as a negative control, and the experiment was repeated in triplicate.

### 2.7. Evaluation of Cytotoxic and Anticancer Activities of GNPs

#### 2.7.1. Assessment of Cytotoxicity

To assess the cytotoxic effects of GNPs against cancerous (HeLa; human cervical cancer cells) and normal human primary osteoblasts, MTT cytotoxicity colorimetric assay was performed [36]. Briefly, the cells (1 × 10^4^ cells per well) were plated in a 96-well plate and incubated for 24 h at 37 °C. The cell lines were treated with GNPs at different concentrations ranging from 0.62 to 20 µg/mL in triplicates and incubated for 48 h. Ten μL of the MTT reagent (0.5 mg/mL) was then added to each well and the plates were further incubated for 4 h. Then, 150 µL DMSO was added to each well to solubilize formazan crystals. Cell viability was quantified by measuring the optical densities (OD) at 570 nm with a reference filter of 655 nm using a Microplate Reader (BIORAD-680). The untreated cells served as controls and the results are presented as percentage of viable cells compared with the control.

#### 2.7.2. Analysis of Cytomorphological Changes in HeLa

HeLa cells were pretreated GNPs (at their IC_50_) and were incubated at 37 °C in 5% CO_2_. At 48 h post incubation, gross morphological changes in the cells were observed using an inverted phase contrast microscope (Nikon ECLIPSE Ti-S, Tokyo, Japan).

#### 2.7.3. Detection of Nuclear Condensation

DAPI (4′, 6-diamidino-2-phenylindole), a fluorescent nuclear dye was used to assess the apoptotic potential at IC_50_ concentrations of GNPs on HeLa cell line [36]. An inverted fluorescence magnifying microscope (Nikon ECLIPSE Ti-S, Japan) was utilized to capture images of stained cells. The intensities of fluorescence in treated HeLa cells with GNPs in IC_50_ in comparison to untreated cells were measured by J-image program. The mean signal intensities and standard deviations for at least 50 treated cells were calculated.

#### 2.7.4. Evaluation of Intracellular Reactive Oxygen Species (ROS) Production

In order to detect the effect of GNPs on ROS production, treated and untreated HeLa cells with GNPS in IC_50_ for 48 h at 37 °C were stained with fluorogenic reagent H2DCFDA [39]. An inverted fluorescence magnifying microscope (Nikon ECLIPSE Ti-S, Japan) was used. The intensities of fluorescence in GNPs (IC_50_) treated HeLa cells in comparison to untreated cells were measured by the J-image program. The mean signal intensities and standard deviations for at least 50 treated cells were calculated.

## 3. Results

### 3.1. Biosynthesis of GNPs

In this study, *B. hispida* was used as both a reducing and stabilizing agent, and HAuCl_4_ (1 mM) acted as the gold precursor. It was proposed that aqueous peel extract triggers the formation of GNPs by the aid of its reducing enzymes as well as capping agents like secondary metabolites, which synergistically reduce AuCl_4_ (+3 oxidation state) into Au (0 oxidation state). The reduction of HAuCl4 was visibly detected by color change of *B. hispida* peel extract from green into ruby red color, confirming the formation of GNPs.

### 3.2. Characterization of the Prepared GNPs

#### 3.2.1. UV–Visible Spectroscopy of Synthesized GNPs

**Figure 1:**
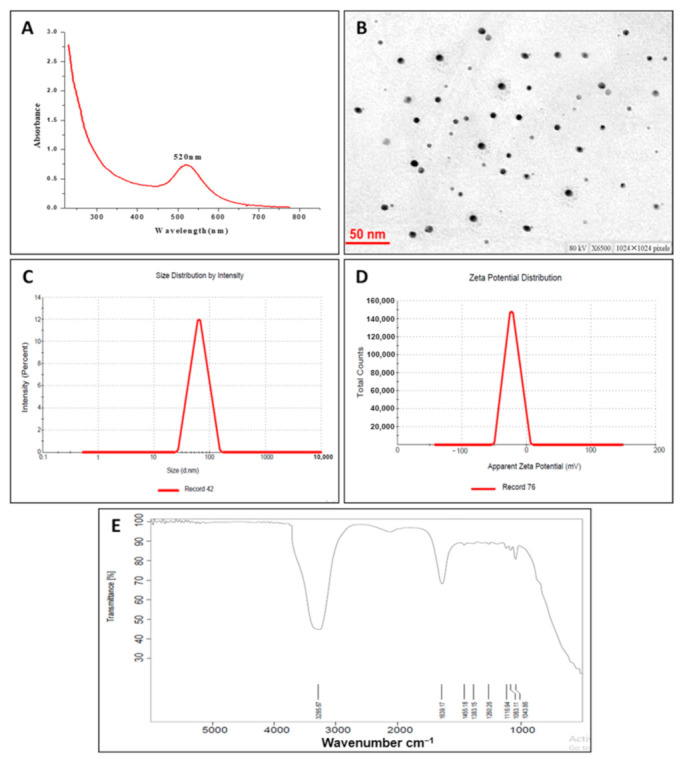
Characterization of gold nanoparticles (GNPs): (**A**) UV–VIS spectroscopy, (**B**) transmission electron microscopy, (**C**) dynamic light scattering, (**D**) zeta potential, and (**E**) Fourier transform infrared (FTIR) spectrum.

The surface plasmon resonance (SPR) represents a peculiar phenomenon to noble metal nanoparticles that contributes to intense electromagnetic fields on the particle surface, which in turn increases all radiative properties such as scattering and absorption [40]. Herein, therefore, the formation of GNPs was confirmed by UV–VIS spectra (Figure 1A). Sharp absorption peaked at 520 nm, corresponding to the surface plasmon resonance (SPR) band of the GNPs [41]. However, no obvious peak was observed for *B. hispida* peel extract.

#### 3.2.2. Particle Size and Zeta Potential

The average particle size and particle size distribution profile of the prepared GNPs were determined using the dynamic light scattering (DLS) technique. As shown in Figure 1B, GNPs had an average particle size of 70 nm with a polydispersity index (PDI) of 0.219, indicating homogenous size distribution. The zeta potential of GNPs was also investigated (Figure 1C). Generally, a zeta value of ±20 mV is needed for colloidal stability of nanoparticles [42]. The zeta potential of the prepared GNPs was −26 mV, indicating high stability of the particles. No signs of agglomeration or clumping were observed in the aqueous dispersion of GNPs upon storage at room temperature, presumably due to the electrostatic repulsive forces between the nanoparticles, which hinder nanoparticles from getting closer to each other.

#### 3.2.3. Transmission Electron Microscopy (TEM) Analysis

TEM analysis was adopted to investigate the morphology, shape, and size of GNPs. TEM micrographs (Figure 1D) depicted that GNPs were spherical in shape and uniformly distributed without significant agglomeration. The average size of GNPs determined by TEM was 22.18 ± 2 nm, which was comparatively smaller than that determined by the DLS technique. The particle size estimated by TEM represents the exact diameter of particles as measured in the dry state, while the size determined by the DLS technique is a hydrodynamic diameter (hydrated state). Consequently, the particles will show larger hydrodynamic volume due to the solvent effect in the hydrated state [43].

#### 3.2.4. Fourier Transform Infrared (FTIR) Spectroscopy Analysis

FTIR spectroscopy was performed to determine the potential functional groups in the synthesized GNPs (Figure 1E). The spectra of GNPs showed characteristic absorption peaks at 1639 cm^−1^ corresponding to C=O groups. A medium-wide shoulder, corresponding to amide I linkage and amide II band, was observed at 1455 cm^−1^ due to carboxyl stretch and N-H twist in the amide bond of the proteins, which were capped, or surface modified on the GNPs [44]. The peak at 3285 cm^−1^ was due to the N–H stretch vibration, which depends on the strength of hydrogen bonding rather than backbone confirmation [45]. In addition, ether and alcohol groups (C-O-C/C-OH) C-O stretching, and C-N (aliphatic amine) stretching vibration were observed at 1083 cm^−1^.

### 3.3. Antibacterial Activity of GNPs

**Figure 2:**
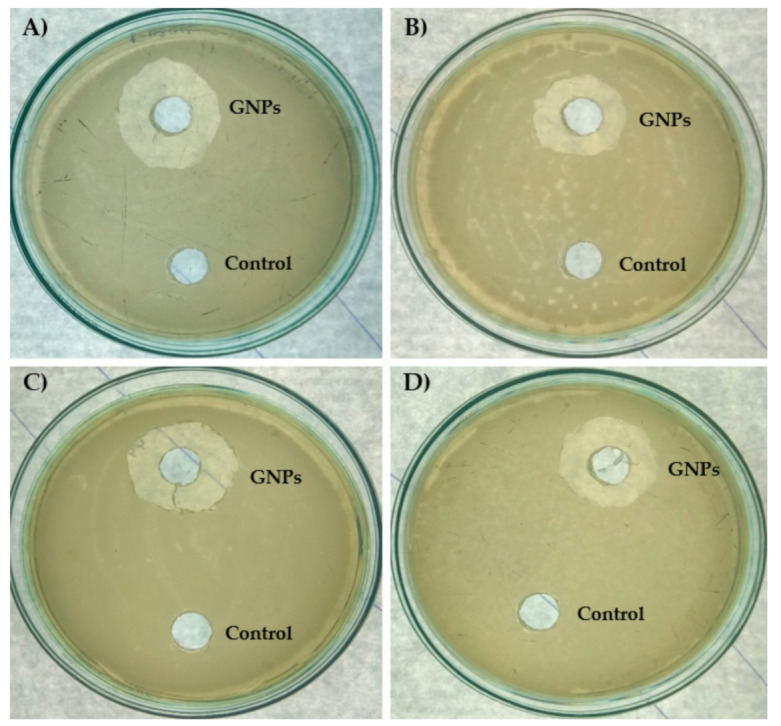
Qualitative assessment of antibacterial activity of GNPs. Müeller–Hinton (MH) agar plates were seeded with standardized suspensions (equivalent to the 0.5 McFarland) of (**A**) *Escherichia coli*, (**B**) *Staphylococcus aureus*, (**C**) *Salmonella abony*, and (**D**) *Klebsiella pneumonia*. Equal amounts of GNPs and PBS (negative control) were poured in the wells made in MH plates. After overnight incubation at 37 °C, inhibition zones around wells of GNPs (10 µg/mL) against all tested bacterial species, in comparison to control, were observed.

**Figure 3:**
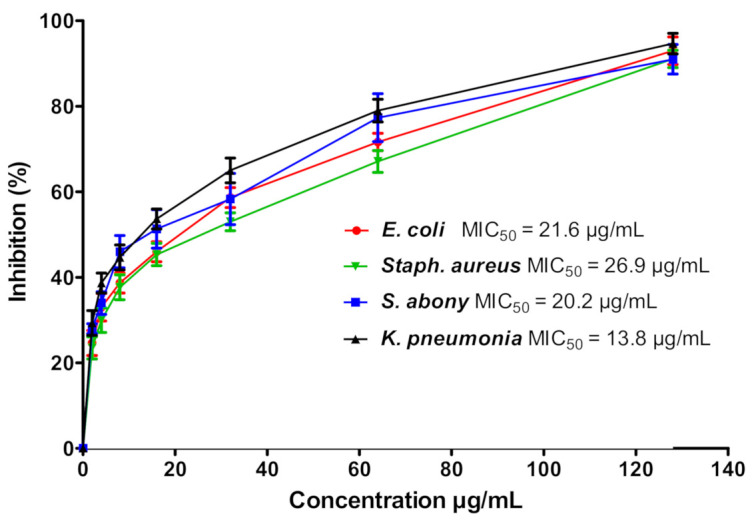
Determination of minimum inhibitory concentration (MIC) of GNPs. Aliquots of GNPs were serially diluted in 96-well microtiter plates in tryptic soy broth (TSB) medium. Aliquots (10 μL) from prepared standard suspensions (equivalent to the 0.5 McFarland) of tested bacterial strains were added to each well. After overnight incubation at 37 °C, the bacterial cells were collected and viably counted. The experiment was repeated in triplicate and the data shown are the means ± standard errors. The MIC was the lowest concentration of GNPs that completely inhibited the bacterial growth, and MIC_50_ was the GNPs concentration that inhibited 50% of the bacteria.

To ensure the antibacterial activities of GNPs, it was tested against various bacterial strains Gram-negative *Escherichia coli* (*E. coli*), *Salmonella abony* (*S. abony*), and *Klebsiella pneumonia* (*K. pneumonia*) and Gram-positive *Staphylococcus aureus* (*S. aureus*). The tested strains were selected to represent different bacterial machineries that harbor different arsenals of virulence factors, besides their noticeable pathogenies and high prevalence in our life [46,47]. Initial findings revealed the ability of GNPs to diffuse in agar and inhibit bacterial growth (Figure 2). GNPs inhibited the growth of *E. coli*, *S. abony*, *K. pneumonia*, and *S. aureus* at concentrations MIC_50_ 21.6, 20.2, 13.8, and 26.9 μg/mL, respectively (Figure 3). Furthermore, the MBC values of GNPs against *E. coli*, *S. abony*, *K. pneumonia*, and *S. aureus* strains were determined to be 80.8, 84.5, 65.5, and 111.5 μg/mL, respectively. Similar findings were shown by Soliman et al. who verified the potent antibacterial activity of silver nanoparticles prepared by aqueous *B. hispidia* extract against different pathogenic bacteria [36]. Nevertheless, it appeared that silver nanoparticles exerted superior antibacterial activity against different pathogenic bacteria, compared to GNPs. The enhanced antibacterial activity of silver nanoparticles might be ascribed to the inherent antimicrobial properties of silver ions, compared to gold ions [48].

Antimicrobial resistance is one of the most challenging global public health issues due to the limitation in the therapeutic options for those infections [4,47]. Many reports tackle the problem of bacterial resistance by renewal of the therapeutic applications of the medicinal plants or drug repurposing [4,46,47]. Moreover, new approaches were developed to improve the distribution, penetration, targeting, and pharmacokinetics of antimicrobial drugs; one of these is drug nanoparticle formulations [36,38]. Metallic nanoparticles were employed for delivering antimicrobials efficiently, showing a magnificent enhancement in targeting and improvement of pharmacokinetics [5,6,8,9,28,49]. Moreover, metallic nanoparticles were shown to have antimicrobial activities, and efficiently synergized the antimicrobial activity of natural product [6,13,14].

Gold has been appreciated as the inorganic antibacterial agent of choice to combat infections and spoilage since ancient times. In the current study, GNPs inhibited a broad spectrum of bacteria, this owed to several mechanisms. In addition to high penetrative power of GNPs, they target bacterial cell membrane, cell wall, DNA, and proteins [12,24,36,50]. It has been repeatedly reported that GNPs can generate pits in bacterial membrane and cell wall. It was shown that GNPs can target cell membrane subcellular compartments, creating pits and leading to cellular decomposition and death. Furthermore, GNPs destroy the glycan N-acetylglucosamine and N-acetylmuramic acid linkage and create a link between the peptide surface and glycan ports of the cell wall, resulting in pit generation in cell walls [51]. In addition to cidal targeting of GNPs to cellular membrane and cell wall, GNPs have been demonstrated to target more bacterial targets as respiratory chain dehydrogenases and bacterial chromosome [9,12,52]. Moreover, the ability of metallic nanoparticles to release reactive oxygen species (ROS), which slow the oxidation of liberated gold ions, confers an additional biocidal activity [9,53].

### 3.4. GNPs Cytotoxicity and Anticancer Activity

**Figure 4:**
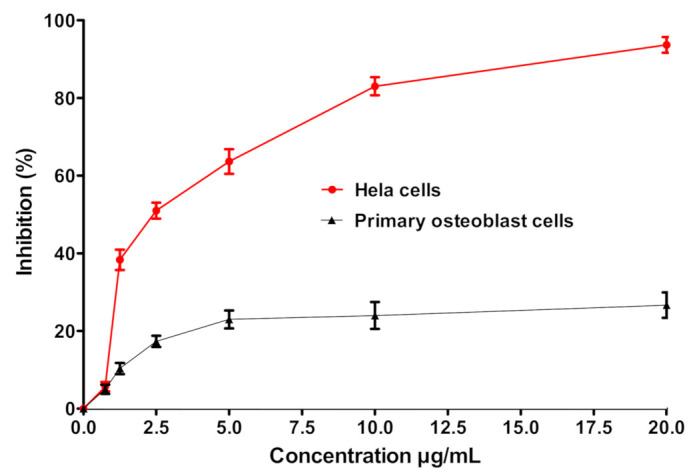
Assessment of GNPs cytotoxicity and determination GNPs IC_50_ on cancer cells. Normal human primary osteoblasts and human cervical cancer cell (HeLa cells) were plated overnight at a density of 1 × 10^4^ cell per well in a 96-well plate at 37 °C. The normal or cancer cells were treated with different concentrations of GNPs and the in vitro cytotoxicity was evaluated using MTT assay. The inhibition percentages were calculated relative to negative control and IC_50_ was the GNPs concentration, which inhibits 50% of HeLa cells. The experiment was conducted in triplicate and the data shown are the means ± standard errors.

In this study, the toxic effects of GNPs were evaluated on cancer HeLa cells and normal osteoblasts cell line using MTT assay. The cell viability was screened at different GNPs concentrations (0.62, 1.25, 2.5, 5, 10, and 20 μg/mL). As shown in Figure 4, HeLa cells rapidly lose their viability upon incubation with GNPs at a concentration range of 0.62 to 20 µg/mL. The estimated IC_50_ value of GNPs on HeLa cells was 2.25 µg/mL. Of interest, GNPs were found to be less cytotoxic against primary osteoblasts, even at higher acceptable biological limit (20 µg/mL). Cancer selectivity of GNPs might be ascribed to the preferential uptake of GNPs by cancer cells compared to normal cells, apparently due to atypical metabolism and increased proliferation rate of cancer cells, compared to the longer doubling time of normal osteoblasts (>96 h) [54,55]. Similar findings were reported by Soliman et al. who emphasized the potent cytotoxic effect of silver nanoparticles prepared by aqueous extract of *B. hispida* against HeLa cancer cells, compared to normal osteoblasts [36].

**Figure 5:**
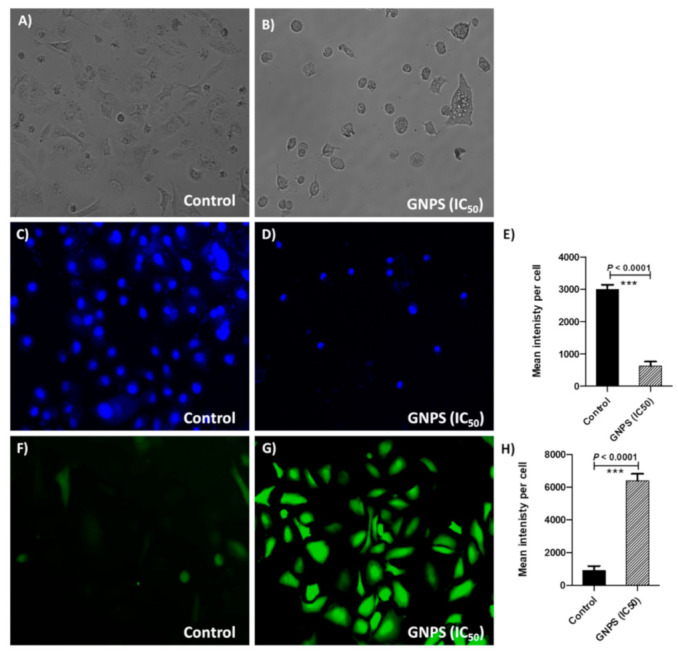
Evaluation of anticancer activity of GNPs. (**A**,**B**) Changes in cellular morphology: HeLa cells were pretreated with PBS (negative control) or GNPs in IC_50_ for 48 h at 37 °C in 5% CO_2_ atmosphere and the morphological changes were observed. (**C**,**D**) Changes in nuclear morphology: the nuclei of untreated control or treated HeLa cell with GNPs at IC_50_ were stained with fluorescent nuclear dye DAPI. (**E**) The mean signal intensities and standard deviations for at least 50 DAPI stained treated or untreated HeLa cells with GNPs at IC_50_ were measured. (**F**,**G**) Intracellular reactive oxygen species (ROS) production: untreated (control) or treated HeLa cells with GNPs at IC_50_ were stained with fluorogenic reagent H2DCFDA. (**H**) The intensities of fluorescence in GNPs (IC_50_) treated HeLa cells in comparison to untreated cells were measured; the mean signal intensities and standard deviations for at least 50 treated cells were calculated.

HeLa cells with 70% confluence were co-incubated with or without GNPs at IC_50_ concentration for 48 h. The phase-contrast microscopic images exhibited morphological changes in HeLa cells (Figure 5B) in contrast to control untreated normal cells (Figure 5A). Several notable changes in shape (turning into circular), loss of membrane integrity, clumping of cells, condensation of cytoplasm, and inhibition of cells growth were observed in treated HeLa cells.

It is widely recognized that nanomaterials could efficiently interact with cancerous cells [6,14]. Many reports have demonstrated the impact of particle size, morphology, surface charge, functional surface modification, and different types of cells on cellular uptake of GNPs and their subcellular distribution [56,57,58]. Furthermore, various strategies have been established, exploiting the physiochemical properties of GNPs themselves (passive targeting) or employing active targeting moieties to ensure efficient intracellular delivery of GNPs to cancer cells [59,60]. In this study, the mode of cellular internalization and, subsequently, the interaction with nuclear material, were evaluated by utilizing a DAPI fluorescent dye (Figure 5C,D). HeLa cells treated with or without GNPs (at IC_50_ value) were incubated for 48 h at 37 ℃ and stained by the DAPI dye. Compared to untreated cells, HeLa cells treated with GNPs induced potent apoptotic effects as manifested by condensed chromatin and dark blue fluorescent consolidated nuclei, which might be ascribed to the expanded cell membrane penetrability [61]. The experiment was conducted in triplicate and the fluorescence intensities were measured; Student’s t-test (Graphpad Prism 8 software) was used to compare between the fluorescence intensities in HeLa cells treated or untreated with GNPs in IC_50_ (Figure 5E). Significantly, the fluorescence intensities were decreased (*p* < 0.0001) in treated cells in comparison to untreated cells, indicating GNPs apoptotic effects due to consolidation of HeLa cells’ nuclei.

ROS-mediated toxicity is one of the mechanisms by which nanomaterials act as anticancer agents [3,7,10,12]. The generated oxidative pressure due to overproduction of ROS leads to enhancement of apoptosis, resulting in considered anticancer activity of GNPs [6,9,50]. In the current study, the intracellular ROS generation in HeLa cells treated with GNPs, was estimated using an oxidation-sensitive fluorogenic marker, H2DCFDA, (Figure 5F,G). Intense fluorescence signals, along with vandalized morphological structure, were observed in HeLa cells treated with GNPs, indicating ROS induced plasma membrane disruption. On the other hand, control (untreated) cells did not show any remarkable fluorescence and retained their natural morphology. The experiment was conducted in triplicate and the fluorescence intensities were measured; Student’s t-test (Graphpad Prism 8 software) was used to compare between the ROS production in HeLa cells treated or untreated with GNPs in IC_50_ (Figure 5H). The production of intracellular ROS was significantly increased in HeLa cells treated with GNPs in IC_50_ compared to untreated cells. GNPs ability to penetrate and efficiently distribute inside cancer cells targeting their genomic content was documented [23,24,25,50]. In this study, we found that GNPs were efficiently accumulated in cancer cells’ nuclei and increased the production of ROS, which can induce considerable anticancer activity.

## 4. Conclusions

In the current study, we report a simple one-step ecofriendly method for the synthesis of GNPs using *B. hispida* peel extract as a reducing and stabilizing agent. The formation of GNPs was detected by color change from light green to ruby red and was confirmed by the existence of a characteristic absorption peak at 520 nm. The biosynthesized GNPs were able to inhibit the growth of various Gram-positive and Gram-negative bacterial strains with comparable competency. In addition, GNPs showed potent cytotoxic effect against HeLa cancer cell line, without exerting remarkable toxicity against normal human osteoblast cell line. Such potent cytotoxic effect was linked with a significant destruction of nuclei of cells and an increase in ROS production in cancer cells. Collectively, our results suggest that *B. hispida* peel extract can be efficiently utilized for green synthesis of GNPs, which can be used as a natural antibacterial and anticancer agent. However, further studies are warranted to evaluate the in vivo activities of GNPs prior to offering them as a broad platform in the field of medicine, either alone or as carriers to other drugs.

## Data Availability

The data presented in this study are available on request from the corresponding author.
